# A knockout mutation associated with juvenile paroxysmal dyskinesia in Markiesje dogs indicates *SOD1* pleiotropy

**DOI:** 10.1007/s00439-021-02271-6

**Published:** 2021-03-07

**Authors:** P. J. J. Mandigers, F. G. Van Steenbeek, W. Bergmann, M. Vos-Loohuis, P. A. Leegwater

**Affiliations:** 1 Department of Clinical Sciences, Faculty of Veterinary Medicine, Utrecht University, PO Box 80154, 3508 TD Utrecht, The Netherlands; 2 Department Biomolecular Health Sciences, Faculty of Veterinary Medicine, Utrecht University, Utrecht, The Netherlands

## Abstract

A juvenile form of paroxysmal dyskinesia segregated in the Markiesje dog breed. Affected pups exhibited clinical signs of a severe tetraparesis, dystonia, cramping and falling over when trying to walk. In most cases, the presentation deteriorated within weeks and elective euthanasia was performed. Pedigree analysis indicated autosomal recessive inheritance. Genome-wide association and homozygosity mapping of 5 affected dogs from 3 litters identified the associated locus on chromosome 31 in the region of *SOD1*. The DNA sequence analysis of *SOD1* showed that the patients were homozygous for a frameshift mutation in the fourth codon. None of the other analyzed dogs of the breed was homozygous for the mutation, indicating full penetrance of the genetic defect. Mutations in *SOD1* are known to cause recessive degenerative myelopathy in middle-aged dogs with low penetrance and dominant amyotrophic lateral sclerosis in humans with variable age of onset. Our findings are similar to recent observations in human patients that a loss of function mutation in *SOD1* leads to a juvenile neurologic disease distinct from amyotrophic lateral sclerosis.

**Supplementary Information:**

The online version contains supplementary material available at 10.1007/s00439-021-02271-6.

## Introduction

Paroxysmal dyskinesias (PDs) are a group of diseases of the central nervous system that produce abnormal movements occurring involuntary and in episodes (Urkasemsin et al. 2014; Lowrie et al. 2017). The duration of these episodes can vary from seconds to hours with a sudden start and ending (Lowrie et al. 2017). The observed abnormal and involuntary movements can comprise athetosis, dystonia, ballismus/hemi-ballismus and chorea (Lowrie et al. 2017). Between the episodes, motor functions are normal and there are no neurological deficits, despite the visible dramatic clinical signs during an attack. Paroxysmal dyskinesias have been described in several dog breeds with variable clinical presentations and outcomes (Packer et al. 2010; Black et al. 2014; Gill et al. 2012; Lowrie et al. 2016; Kolicheski et al. 2017; Polidoro et al. 2020). Breed predisposition points to an inherited origin and causative mutations have been found in *BCAN* in the Cavalier King Charles Spaniel with episodic falling (OMIA 001592–9615) and in *PIGN* in Soft Coated Wheaten Terriers with PD (OMIA 002084–9615; Gill et al. 2012; Kolicheski et al. 2016). In Malteser dogs and Border terriers, PD is most likely induced by gluten and hence called paroxysmal gluten-sensitive dyskinesia (Stassen et al. 2017; Lowrie et al. 2018; Polidoro et al. 2020). The Markiesje is a small Dutch breed of middle-sized dogs that is only recognized by the Dutch Kennel Club. Since 2003 occasionally litters were born with pups that exhibited clinical signs of a severe, rapidly progressing PD. Any effort to move seemed to trigger an episode. Here we describe the clinical presentation of the disorder and the molecular genetic study to identify its origin.

## Materials and methods

### Dogs

Routine physical and neurological examinations by a board-certified European Specialist in Veterinary Neurology (PJJM) were performed on 13 Markiesje pups that were presented to the Utrecht University Clinic for Companion Animals with signs of a paroxysmal dyskinesia. Additional examinations pertained to hematology, clinical chemistry, urinalysis, and radiography of the vertebral column. If possible an EMG (electromyogram), tensilon test and additional blood and urine examination (for myasthenia gravis, toxoplasmosis, neospora, and organic acid analysis) was performed as well. The tensilon test consists of the intravenous injection of the drug edrophonium that blocks the degradation of acetylcholine in the neuromuscular junction. If the clinical signs diminish or vanish, the disease most likely is myasthenia gravis.

If the owners elected euthanasia a post-mortem examination was performed by a board-certified European Specialist in Veterinary Pathology (WB). DNA was available of 6 affected Markiesje dogs from 4 litters, 8 unaffected siblings, and 7 parents and 1 grandparent (Supplementary Figure S1). Unrelated dogs from the same breed were sampled at a club match and samples were submitted for DNA testing by individual owners. DNA was isolated from EDTA blood samples using a Chemagic MSM1 robot (Perkin Elmer).

### Molecular genetic analysis

Five affected and 18 unrelated Markiesje dogs were genotyped with the Illumina CanineHD SNP array with over 170,000 SNPs. Genome-wide association study was performed with PLINK version 1.07 software (Purcell et al. 2007). The region that displayed strong association was inspected in the output file allelicassoc.assoc for shared homozygosity by the cases. Four SNPs were selected from the region to genotype a sixth patient, healthy siblings, parents and a grandparent. The DNA sequences of oligonucleotides used for PCR and chain termination reactions are listed in Supplementary Table S1. Coincidently, additional informative SNPs were located on two of the PCR fragments, at CanFam3.1 positions g.31:25160489 and g.31:25644241. The PCR conditions and procedures for DNA sequence analysis were as described elsewhere (Wu et al. 2020a).

Enrichment of protein-coding gene exons of the chromosome region of interest from genomic DNA and library preparation for next-generation sequencing (NGS) was as described (Wu et al. 2020b). The positions of the exon sequences used for enrichment are listed in Supplementary Table S2. The NGS was performed on a MiSeq according to the protocol of the manufacturer (Illumina). Reads of 151 bp were collected as mate pairs. The NGS data quality control was as described (Wu et al. 2020b) and variants were annotated using SIFT (Kumar et al. 2009).

The gene of interest, *SOD1*, was further analyzed by conventional DNA sequencing using BigDye v.3.1 of Applied Biosystems (Thermo Fisher). The exons of the gene were amplified and subsequently sequenced with primers described by others (Zeng et al. 2014). The DNA sequences were generated by a Genetic Analyzer 3130xL (Applied Biosystems) and analyzed with Seqman Pro 14 software of the Lasergene package (DNASTAR). LOD scores for linkage were calculated using Superlink version 1.7 (Fishelson and Geiger 2004).

## Results

### Clinical presentation

From 2003 to 2016, 14 young Markiesje dogs, born in 7 litters that consisted of a total of 36 pups, were presented with clinical signs of a severe tetraparesis, dystonia, and cramping. All cases but one were examined by one of us (PJJM). The first signs became manifest at an age of around 10 weeks. They fell over when the owners tried walking them (Supplementary Video S1). At rest, a physical and neurological examination did not reveal any abnormalities but when stressed or walked the pups started to show cramping and dystonic features of all legs. In all pups, routine hematology and clinical chemistry and urinalysis did not reveal any significant abnormalities. In two pups the complete vertebral column was radiographically examined, in four pups an EMG (electromyogram) performed and these did not reveal any abnormalities. On four pups a so-called tensilon test was performed that ruled out myasthenia gravis. In two pups the acetylcholinesterase receptor titer for myasthenia gravis was measured and was found not to be abnormal. In seven pups titers for toxoplasmosis and neospora revealed no increase. In three pups an additional organic acid analysis was performed, which did not reveal any abnormality. All 7 mothers of affected dogs and 10 siblings were examined and none of these displayed clinical signs of a movement disorder.

In attempts to alleviate the clinical signs, the pups were treated with drugs such as non-steroidal anti-inflammatory drugs, phenobarbital, phenytoin, benzodiazepines, acetazolamide, pyridostigmine, and corticosteroids, but none of the pups showed any improvement. Although at rest the pups showed limited clinical signs their ability to function normally was extremely limited and twelve owners elected euthanasia before the age of 3 months. Routine post-mortem examinations were possible in 8 of these 12 pups. These revealed no morphological abnormalities macroscopically. Histologically, the most consistent finding was mild, random Wallerian-like degeneration in the brain stem and spinal cord with mild denervation atrophy of the skeletal muscle. Two owners continued to take care of the affected pups and although they were severely handicapped, one dog lived for three years after diagnosis and the other for 10 years after diagnosis.

### Gene mapping and analysis

**Fig. 1:**
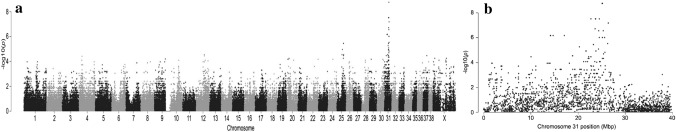
Genome-wide association study of paroxysmal dyskinesia in Markiesje dogs. **a** Manhattan plot of the SNP-data of 5 cases and 18 unrelated controls indicated the involvement of chromosome 31. **b** Zoom of chromosome 31 points to the region around position 25 Mbp

Markiesje dogs with PD were born from healthy parents that often were related within a limited number of generations (Supplementary Figure S1). Analysis of the pedigrees of all affected dogs suggested that the disease followed Mendelian inheritance and was autosomal recessive. The genome-wide association analysis of 5 affected Markiesje dogs and 18 unrelated controls of the same breed indicated the involvement of a region on canine chromosome 31 (Fig. 1). The *p*-value of 1.8 × 10^–9^ of the three top SNPs was 3 orders of magnitude smaller than that of the next best peak. The top SNPs were located between positions 25.1–25.2 Mbp of CFA31 (CanFam3.1). Inspection of the SNP allele frequencies in the group of affected dogs showed that these shared a region of homozygosity from positions 24.7–26.7 Mbp. A newly presented sixth patient and available siblings and parents were genotyped for six SNPs in the region (Supplementary Figure S1). Only the affected dogs were homozygous for the associated haplotype.

The 2 Mbp region contained few notable protein-coding genes including *GRIK1*, *CLDN8*, and *CLDN17*, 15 genes for keratin-associated proteins, *TIAM1*, *SOD1*, and *SCAF4*. Of these, only mutations in *SOD1* were known for well-established neurologic phenotypes in humans and dogs: amyotrophic lateral sclerosis type 1 (ALS1, OMIM 105400) and progressive spastic tetraplegia and axial hypotonia (OMIM 618598), and degenerative myelopathy (DM, OMIA 000263–9615), respectively. We considered this gene, located at 26.5 Mbp of CFA31, the prime candidate gene for involvement in PD in the Markiesje dogs.

Targeted NGS analysis of the exons of protein-coding genes in the CFA31 region of 24.9–26.7 Mbp of 3 cases and 2 unrelated controls did not yield a variant as the likely cause of PD in Markiesje dogs (Supplementary Table S3). A predicted high impact variant was detected at position 31:g.26539786 in the annotated exon 1 of *SOD1* of a control dog. However, this exon does not align with the reference cDNA sequence of canine *SOD1*. The proper exon 1 is located in a gap of the reference genome sequence and was, therefore, not included in the NGS analysis.

**Fig. 2:**
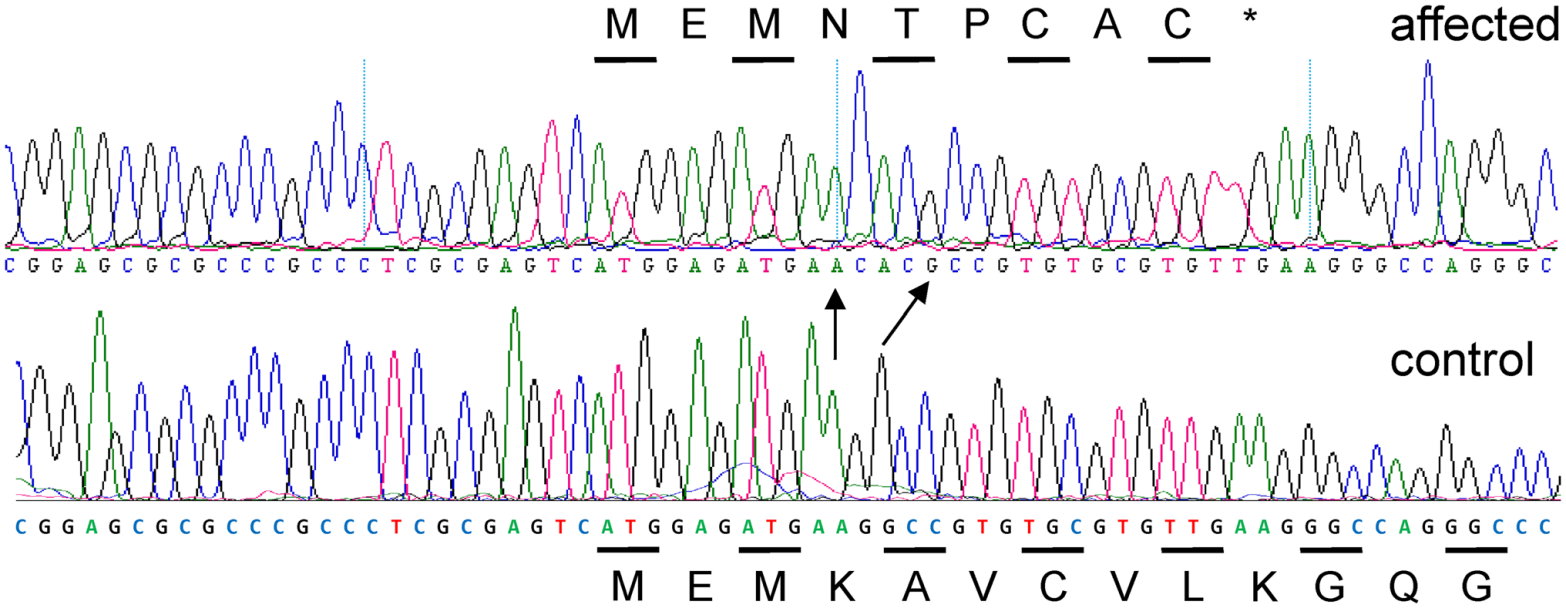
DNA sequence of the translation start region of exon 1 of *SOD1* in Markiesje dogs with and without paroxysmal dyskinesia. In the affected dog, the first of two G-residues is replaced by the trinucleotide CAC. The arrows indicate unaltered A- and G-residues. The mutation leads to a frameshift at the 4th codon of the gene. The codons are indicated by alternating lines with the encoded amino acid in one letter code. *Denotes the TGA stop codon

The coding DNA sequence of *SOD1*, including the correct exon 1 sequence, was analyzed by conventional techniques and we identified a frameshift mutation in the analyzed affected Markiesje dogs (Fig. 2). A G-nucleotide of the fourth codon of the gene is replaced by a CAC-trinucleotide. The shifted coding sequence runs into a stop codon at the tenth codon. The annotation of the indel mutation is NM001003035.1:c.12delinsCAC. The protein annotation is NP_001003035.1:p.Lys4Aspfs*6. The genomic annotation cannot be given because exon 1 of *SOD1* is not represented in the reference genome CanFam3.1. Genotyping of available Markiesje dogs from the pedigree showed that only the affected dogs were homozygous for the mutation (Supplementary Figure S1). The LOD score for linkage between the mutation and PD in the pedigree of the six affected dogs was 4.5.

Of the Markiesje dogs from the general population, 31 out of 184 dogs were heterozygous and none were homozygous for the *SOD1* frameshift mutation, corresponding to an allele frequency of 0.08, which is not unusually high in dog genetics.

## Discussion

We report a new inherited neurologic disorder that occurred in juvenile dogs of the Markiesje breed. Based on the clinical presentation, the disorder is classified as a paroxysmal dyskinesia. Never before has PD been described in pups of this age with such a poor clinical outcome. Typical characteristics of PDs are its sudden presentation, its sudden disappearance and the absence of autonomic signs. During the events, that can, in contrast with epilepsy, last for minutes to hours, all pups remained conscious and for this reason a (focal) epilepsy was ruled out. Most dogs with a PD have a low frequency of events and can be clinically managed rather reasonably. Dogs that show many PD events are treated accordingly and rarely euthanasia is elected. In contrast, in the affected Markiesje pups the PD was almost always present when the dogs were stressed or walked. For this reason, the possibility of disorders such as myopathies and myasthenia gravis were carefully examined and although the outcome was negative, the treatment was based on these disorders. None of the pups responded to the treatment, suggesting that this group of differential diagnosis was also not applicable.

Paroxysmal dyskinesias are by itself rarely lethal. Maybe this is also the case in this breed as two of the 14 pups were not euthanatized after diagnosis and these pups continued to live, although severely handicapped, for several years. For all these reasons we classify the neurological disorder in these pups as a PD. In human medicine, PDs are classified into Paroxysmal Kinesigenic Dyskinesia in which episodes are provoked by movements that occur suddenly; Paroxysmal Non-Kinesigenic Dyskinesia in which movements are not the cause of the episodes but alcohol, stress, caffeine and fatigue may be triggers, and Paroxysmal Exertion- Induced Dyskinesia with continuous exercise as precipitating factor (Lowrie et al. 2017). In veterinary medicine most PDs are either classified as a Paroxysmal Gluten-Sensitive Dyskinesia or as an unspecified Paroxysmal Dyskinesia. It is difficult to differentiate the disease in dogs further because often they show signs that warrant classification in each of the human types of PD. In the Markiesje breed one could argue that the disorder resembles a Paroxysmal Kinesigenic Dyskinesia.

We were able to identify the gene mutation that most likely causes the disease. The LOD score of 4.5 for linkage, well above the threshold value of 3, is proof for the involvement of the *SOD1* gene region. The mutation identified in *SOD1* leads to a frameshift after the third codon, effectively knocking out the gene. In dogs, two point mutations in *SOD1* have been established as the cause of DM in a variety of breeds (Zeng et al. 2014). This disease is characterized by late-onset paralysis due to degeneration of white matter of the spinal cord. The degeneration is accompanied and probably triggered by aggregates of mutant SOD1 protein (Awano et al. 2009). In this respect, DM is a model for ALS1 in humans which is characterized by the death of motor neurons in the brain, brainstem and spinal cord due to *SOD1* mutations, often with SOD1 aggregates. Most forms of ALS1 follow dominant inheritance, although recessive forms have also been described. In general, DM inherits recessively and the frequency of the causative E40K mutation of SOD1 can be quite high in some breeds. The penetrance of DM in dogs with the homozygous genotype is incomplete, however, and varies per breed (Awano et al. 2009; Zeng et al. 2014). Aggregate formation has been found in heterozygous dogs without clinical signs and affected heterozygous cases have been identified (Awano et al. 2009; Zeng et al. 2014). So, DM, such as ALS1, is caused by a gain of function mutation of SOD1, the function gained being aggregate formation (Awano et al. 2009). In contrast, PD in Markiesje dogs is caused by a loss of function mutation of *SOD1* with full penetrance, and it presents as a distinct disease entity. The gene encodes superoxide dismutase 1, one of two scavengers of free superoxide radicals, which are damaging by nature.

Recently, two independent human case reports reached a similar conclusion of pleiotropy of the *SOD1* gene due to distinct pathogenic effects of aggregate formation and loss of superoxide dismutase 1 activity. Two unrelated patients of Afghan descent displayed clinical signs of motor neuron deterioration at ages 6 and 9 months. The disease was termed progressive spastic tetraplegia and axial hypotonia (STAHP, OMIM 618598) and no similarities with ALS1 were recognized. (Andersen et al. 2019; Park et al. 2019). Both patients were homozygous for the same frameshift mutation in *SOD1* after codon 112 and deficiency of SOD1 activity was demonstrated.

The phenotype of the *SOD1* knockout Markiesje dogs is similar to that of the SOD1 deficient human patients. Both diseases manifest at an early age when the juveniles should start moving around. In dog and human patients there is a severe and progressive loss of motor abilities, tetraspasticity predominantly in the lower extremities, and dystonia (Park et al. 2019). The few fasciculations and myokymia observed in one of the human patients were not seen in the Markiesje dogs (Andersen et al. 2019). Cognitive functions seem to be spared.

It can be concluded that *SOD1* mutations are associated with distinct neurologic disease entities in humans and dogs. Deficiency of SOD1 leads to early-onset movement disorders with full penetrance, while mutations that lead to SOD1 aggregation cause motor neuron death with variable and age-dependent penetrance.

## Supplementary Information

Below is the link to the electronic supplementary material.

Supplementary file1 (PDF 106 KB)

Supplementary file2 (PDF 35 KB)

Supplementary file3 (XLSX 16 KB)

Supplementary file4 (MPG 6477 KB)

Supplementary file5 (XLSX 22 KB)

## Data Availability

The NGS data have been deposited in the GEO database and are accessible through GEO Series accession number GSE166712 (https://www.ncbi.nlm.nih.gov/geo/query/acc.cgi?acc=GSE166712).
