# Supplementary material for: A knockout mutation associated with juvenile paroxysmal dyskinesia in Markiesje dogs indicates SOD1 pleiotropy
Source: Hum Genet. 2021 Mar 7;140(11):1547–52. doi: 10.1007/s00439-021-02271-6 (PMC8519843; doi:10.1007/s00439-021-02271-6)
Supplement: Supplementary file 1 — Supplementary file1 (PDF 106 KB) [file 439_2021_2271_MOESM1_ESM.pdf]

Fig.S1

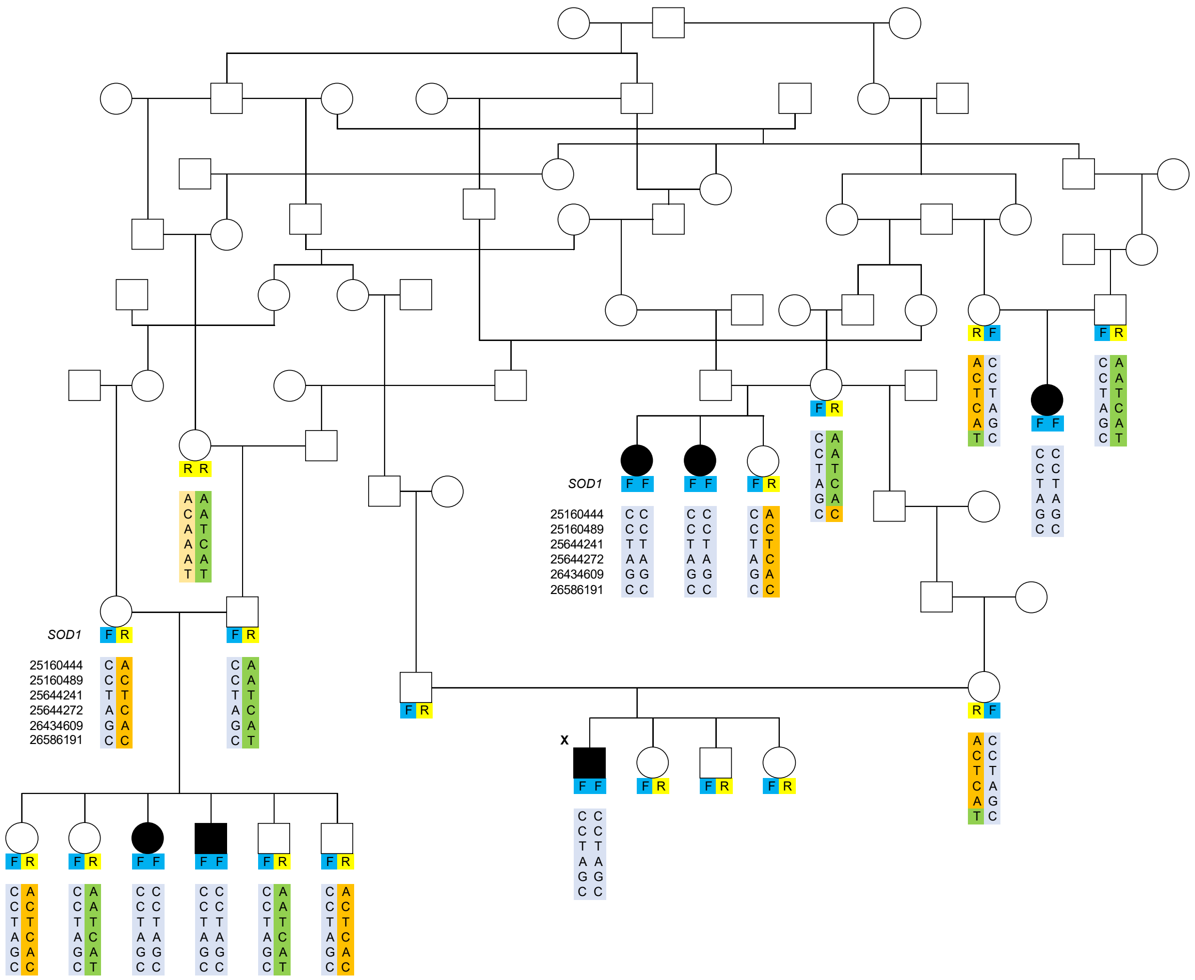

Figure S1. Pedigree and segregation analysis of paroxysmal dyskinesia in Markiesje. The haplotypes were derived of the genotypes of 5 SNPs at indicated positions on chromosome 31 according to CanFam3.1. The grouping of the alleles in colored haplotyes is based on parent - offspring inheritance and on the occurrence of allele combinations in different branches of the pedigree. The x denotes the affected dog that was not included in the genome-wide association analysis. The disease cosegregates with a frameshift mutation (F, blue) in *SOD1*. The *SOD1* reference allele (R, yellow) corresponds to the DNA sequence of Genbank accession NM_001003035.1.
