# Supplementary material for: A knockout mutation associated with juvenile paroxysmal dyskinesia in Markiesje dogs indicates SOD1 pleiotropy
Source: Hum Genet. 2021 Mar 7;140(11):1547–52. doi: 10.1007/s00439-021-02271-6 (PMC8519843; doi:10.1007/s00439-021-02271-6)
Supplement: Supplementary file 2 — Supplementary file2 (PDF 35 KB) [file 439_2021_2271_MOESM2_ESM.pdf]

Supplementary Table S1. Oligonucletides for genotyping of selected Illumina CanineHD SNPs.

CanineHD SNP	CFA31 position[a]	forward primer	reverse primer
BICF2P1156187	25160444	GCTTAACAGTCATAAGTCAGG	TTCTTCTAGTAGCAGCCGAG
BICF2P1103073	25644272	AGGATAAATGTAGCCGATAGGA	TGTAGTTGGAACCTTTAGTGTC
BICF2P755616	26434609	CTTCAGTTTACCTCCATAGCC	CCACCTATGTTATTTAGCACCA
BICF2G630738979	26586191	ACTGTGGTATAGAGAAAGGGA	GACACTGAACTTGATACTTCCT

[a] position on CanFam3.1
